# Fracture Resistance of 3D-Printed Partial and Conventional Veneers

**DOI:** 10.3390/jfb17060298

**Published:** 2026-06-15

**Authors:** Abdulrahman Alshabib, Silvia Rojas-Rueda, Saad Alotaibi, Carlos A. Jurado, Mark A. Antal, Brian R. Morrow, Franklin Garcia-Godoy

**Affiliations:** 1 Department of Restorative Dental Science, College of Dentistry, King Saud University, Riyadh 11545, Saudi Arabia; 2 School of Dental Medicine, Ponce Health Sciences University, Ponce 00732, Puerto Rico; 3 Department of Operative and Esthetic Dentistry, Faculty of Dentistry, University of Szeged, 6720 Szeged, Hungary; 4 Department of Bioscience Research, College of Dentistry, The University of Tennessee Health Science Center, Memphis, TN 382014, USA; morrow@uthsc.edu (B.R.M.);

**Keywords:** 3D-printed, veneers, partial veneers, dentistry

## Abstract

Background: The purpose of this in vitro study was to evaluate and compare the fracture resistance of 3D-printed partial veneers with finish lines at three different locations and conventional full veneers with finish lines at the gingival level. All restorations were digitally designed and 3D printed using a nanoceramic filled resin specifically developed for veneer restorations. Methods: Four maxillary right central incisor typodont teeth were prepared for labial veneers with finish lines at different locations: incisal third (InT), middle portion of the middle third (MmT), lower portion of the middle third (LmT), and conventional veneer with the finish line at the gingival level (CoV). Each preparation was scanned, and 15 casts were 3D printed from each scan. A total of 60 3D-printed veneers were fabricated (n = 15 per group) using a nanoceramic-filled resin designed for veneer restorations. The restorations were cemented to the 3D-printed dies using the manufacturer’s adhesive and resin cement. The specimens were artificially aged with 10,000 thermal cycles between 5 °C and 55 °C, with a dwell time of 30 s, and then loaded to failure using a universal testing machine. Fracture load values were analyzed using one-way ANOVA and the Tukey honestly significant difference post hoc test (α = 0.05). In addition, fracture patterns were evaluated using scanning electron microscopy images for descriptive purposes. Results: The mean fracture resistance of the 3D-printed partial and conventional labial veneers differed significantly depending on restoration design (*p* < 0.05). Among the partial veneers, the LmT group showed the highest fracture resistance (279.86 N), followed by the MmT group (266.92 N), while the InT group showed the lowest value (179.22 N). The conventional veneer group (CoV) demonstrated higher fracture resistance (404.07 N) than all partial veneer groups. Conclusions: The fracture resistance of 3D-printed partial and conventional labial veneers fabricated with nanoceramic-filled resins differed according to finish line location. Conventional veneers demonstrated higher fracture resistance than all partial veneer designs. The smallest partial veneer, with the margin located in the incisal third, showed lower fracture resistance than the partial veneer designs with finish lines in the middle third.

## 1. Introduction

Conservative dentistry emphasizes minimally invasive preparations that maximize the preservation of healthy tooth structure while maintaining function, esthetics, and long-term restorative success. This paradigm shift focuses on removing only compromised or diseased tissue, using adhesive restorative materials, and moving away from conventional full-coverage preparations whenever possible [1,2,3,4]. Preserving enamel and dentin is essential because these tissues contribute to the structural integrity, vitality, and biomechanical behavior of the tooth. Traditional restorative approaches often require substantial tooth reduction to accommodate restorations such as full crowns; however, this may result in unnecessary loss of sound tooth structure, increased susceptibility to pulpal complications, weakened remaining tooth structure, and prolonged treatment time. In contrast, minimally invasive techniques aim to conserve as much natural tooth substrate as possible, allowing restorations to rely on adhesive bonding while reducing biological and mechanical risks. Therefore, these techniques have emerged as progressive and clinically relevant alternatives that prioritize preservation of the natural dentition and support more conservative, patient-centered restorative care [5].

Dental veneers represent a restorative procedure that aligns with the principles of minimally invasive dentistry, as preparation is generally limited to the labial surface compared with conventional crown preparations, which require circumferential tooth reduction [6,7,8]. Previous studies have reported that veneer preparations involve removal of approximately 3% to 30% of coronal tooth structure, whereas traditional full-coverage crowns require removal of approximately 63% to 72% [9]. Furthermore, clinical studies have demonstrated excellent long-term performance of veneer restorations, supporting their use as a reliable, minimally invasive, and durable option in restorative practice [10,11].

The introduction of CAD/CAM dentistry has substantially improved clinical workflows by expediting the fabrication process, enhancing patient comfort, and producing predictable, clinically acceptable restorations [12,13]. Digital workflows reduce several steps associated with conventional impression and laboratory procedures, which may decrease chair time, minimize patient discomfort, and improve communication between clinicians, technicians, and patients. Early CAD/CAM technology allowed clinicians to fabricate only single, relatively simple restorations, such as inlays, onlays, and single crowns; however, current systems enable the fabrication of more complex and multiple restorations with increased precision and efficiency. Nowadays, clinicians can use CAD/CAM workflows to design and fabricate extensive tooth-supported and implant-supported restorations, including multi-unit fixed dental prostheses, full-arch prostheses, and full-mouth rehabilitations. Initially, CAD/CAM dentistry relied on subtractive manufacturing, also known as milling, which significantly accelerated the restorative process compared with conventional techniques such as pressing and lost-wax fabrication [14,15,16]. Chairside CAD/CAM milled restorations can now be completed in a single appointment from start to finish, reducing the need for provisional restorations and multiple clinical visits. In addition, advances in digital scanning, design software, restorative materials, and milling units have improved the accuracy, esthetics, and clinical versatility of CAD/CAM restorations. Clinical reports of milled restorations have demonstrated favorable optical properties capable of fulfilling patients’ esthetic expectations, particularly in the anterior region, where color, translucency, and surface texture are critical for achieving natural-looking outcomes [17,18,19].

Additive manufacturing, also known as 3D printing, represents one of the most recent advancements within the digital dentistry workflow. This technology has expanded the possibilities of digital fabrication by allowing clinicians and dental laboratories to manufacture dental restorations, appliances, and working models directly from computer-aided designs. Unlike subtractive manufacturing, in which restorations are milled from prefabricated blocks or discs by removing excess material, additive manufacturing fabricates objects layer by layer based on a digital design. This layer-by-layer approach reduces material waste, permits efficient fabrication of multiple objects, and enables the production of complex geometries that may be difficult or less efficient to achieve with conventional milling procedures. In tooth-supported restorative dentistry, 3D printing may therefore offer advantages in terms of workflow efficiency, reproducibility, and material conservation, while still allowing the production of restorations with clinically acceptable accuracy. This approach allows dental restorations and appliances to be produced within a relatively short period of time while reducing material waste and enabling the fabrication of complex geometries that may be difficult to achieve with conventional milling procedures [20,21,22].

Initially, 3D-printed materials in dentistry were primarily used for non-definitive applications, such as diagnostic casts, surgical guides, custom trays, and occlusal guards. During the early stages of this technology, limitations related to printing accuracy, surface quality, mechanical strength, wear resistance, and material stability restricted its application mainly to diagnostic and auxiliary procedures. However, continuous improvements in 3D printers, resin formulations, printing resolution, and post-processing protocols have significantly expanded the clinical applications of additive manufacturing. As the technology and material properties improved, printable resin materials were introduced for the fabrication of interim crowns, fixed dental prostheses, and other provisional restorations [23,24]. More recently, advances in printable restorative materials have allowed clinicians to fabricate higher-quality interim restorations and, in selected applications, definitive restorations with improved strength, esthetics, and surface characteristics compared with earlier generations of printable resins. These developments expanded the role of 3D printing from a purely diagnostic and auxiliary tool to a clinically relevant manufacturing method for restorative procedures.

More recently, resin–ceramic hybrid materials have become available for the fabrication of definitive restorations, offering improved mechanical behavior, wear resistance, and esthetic properties compared with earlier printable resins [25,26]. In addition, some 3D-printed nanoceramic-filled resins have been developed specifically for definitive veneer restorations. These materials aim to combine the efficiency and reproducibility of additive manufacturing with enhanced strength, surface quality, and optical properties required for esthetic restorative treatments.

With the advent of minimally invasive techniques and 3D-printing technology, clinicians can now provide 3D-printed veneer restorations. However, limited data are available on 3D-printed veneer restorations fabricated using minimally invasive designs, such as partial veneers with different finish-line locations. Therefore, the aim of this study was to evaluate the fracture resistance of 3D-printed partial veneers with finish margins located in the incisal third, middle portion of the middle third, and lower portion of the middle third. A conventional veneer with a margin at the gingival level was included as the control group. The first null hypothesis was that there would be no difference in fracture resistance among the three partial veneer designs and the conventional veneer. The second null hypothesis was that there would be no difference in fracture resistance among the three 3D-printed partial veneer designs.

## 2. Materials and Methods

**Figure 1:**
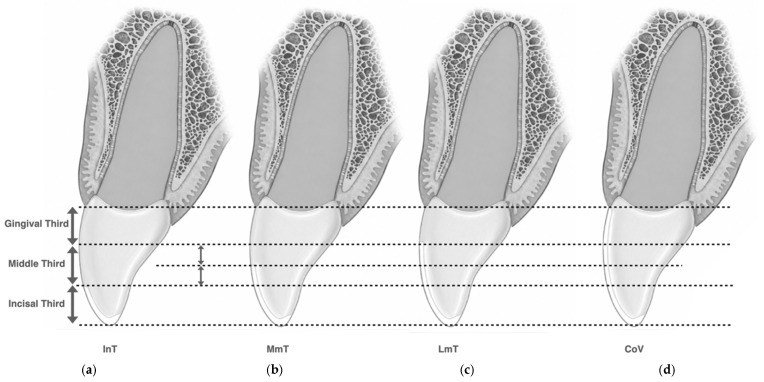
Schematic drawing of the partial and complete veneer restorations. Partial veneer with (**a**) margin in the incisal third (InT); (**b**) with margin in the middle portion of the middle third (MmT); (**c**) with margin in the lower portion of the middle third (LmT); and (**d**) conventional veneer with margin at the gingival level (CoV).

Four maxillary right central incisors (1560 Series; Columbia Dentoform Inc., Lancaster, PA, USA) were prepared for veneer restorations, as follows: partial veneers with finish lines in the incisal third (InT), middle portion of the middle third (MmT), and lower portion of the middle third (LmT), and conventional veneers with the finish line at the gingival level (CoV), which are considered as the control group. All veneer preparations had a 0.3 mm chamfer finish line, 0.5 mm facial reduction, and 0.7 mm incisal reduction (Figure 1).

The prepared typodont teeth were scanned with an intraoral scanner (Primescan; Dentsply Sirona, Charlotte, NC, USA). The scans were exported, and the veneer restorations were digitally designed (exocad DentalCAD 3.1 Rijeka; exocad GmbH, Darmstadt, Germany). A total of 60 restorations (*n* = 15 per group) were 3D printed (SOL; Ackuretta Technologies, Taipei, Taiwan) using a nanoceramic-filled resin (Rodin™ EnVision; Pac-Dent, Brea, CA, USA). The restorations were printed in a vertical 90-degree orientation, with 50 microns of space for the cement. The printed veneer restorations were wiped with 99% isopropyl alcohol using a dampened paper towel (≤5 s contact time) and fully cured for 20 min (Ackuretta Curie; Ackuretta Technologies, Darmstadt, Germany, settings: P7, D2, BL ON). The digital designs of the prepared typodont teeth were modified (Tinkercad; Autodesk, San Francisco, CA, USA) to include a cylindrical base (5 × 10 mm) beneath each preparation. Fifteen dies for each preparation were printed (Form 3; Formlabs, Somerville, MA, USA) using dental cast resin (Model Resin; Formlabs, Somerville, MA, USA). The printed dies were then washed in isopropyl alcohol for 20 min (Form Wash; Formlabs) and post-cured in a UV unit (Form Cure; Formlabs, Somerville, MA, USA) for 30 min at 60 °C according to the manufacturer’s instructions.

The printed dies were first treated with phosphoric acid etching gel (Etchpro; Rodin Envision; Pac-Dent Inc., Brea, CA, USA) for 20 s, then rinsed and air-dried. The intaglio surfaces of the restorations were treated with adhesive (Bond; Rodin Envision; Pac-Dent Inc., Brea, CA, USA); excess adhesive was removed with air, and the restorations were light-cured for 30 s. The veneers were then cemented with resin cement (Veneer Cement; Rodin Envision; Pac-Dent Inc., Brea, CA, USA). Excess cement was removed with a microbrush, and the cemented restorations were light-cured from the incisal, mesial, distal, and facial aspects for 20 s per surface.

The cemented restorations were subjected to artificial aging using 10,000 thermal cycles between 5 °C and 55 °C, with a dwell time of 30 s (Thermocycler THE-1100 SD; Mechatronik, Feldkirchen-Westerham, Germany). The specimens were then placed in a jig and loaded vertically at the incisal edge. A plastic sheet was placed between the incisal edge of the restoration and the loading applicator to simulate food bolus distribution. The specimens were loaded to fracture using a universal testing machine (4411; Instron), fracture load values were recorded in Newtons, and the values from all specimens in each group were averaged. All fractured specimens were visually analyzed, and fracture patterns were identified. One representative specimen per group was selected for evaluation using field-emission scanning electron microscopy (SEM) (ERA 8800FE Elionix; STS Elionix, Tokyo, Japan). Before imaging, a thin layer of gold was applied to the fractured specimen surface using a sputter coater (Quick Coater Type SC-701; Sanyu Electron, Tokyo, Japan) to provide electrical conductivity. Images were obtained at an accelerating voltage of 15 kV.

Data were analyzed using SPSS software (IBM SPSS Statistics, version 30.0, Armonk, NY, YSA). Normality of the data distribution was assessed using the Shapiro–Wilk test. Descriptive statistics were calculated for each group. Homogeneity of variances was evaluated using the Levene test. One-way analysis of variance (ANOVA) was performed to detect differences among groups. Post hoc pairwise comparisons were conducted using the Tukey honestly significant difference (HSD) test. The level of significance was set at α = 0.05.

## 3. Results

The fracture load of 3D-printed partial and complete veneers for maxillary right central incisors fabricated with nanoceramic filled resin is shown in Table 1. The Shapiro–Wilk test indicated normal distribution in all groups (*p* > 0.05) (Table 2). Levene’s test confirmed homogeneity of variances among groups (*p* = 0.318). One-way ANOVA revealed statistically significant differences among the groups (F(3,56) = 949.925, *p* < 0.001). The partial veneer with finish line at the lower portion of the middle third (LmT) displayed the highest values with 279.86 N, followed by partial veneers with finish line at middle portion of the middle third (MmT) with 266.92 N, and the lowest values were displayed by the partial veneers with finish line at the incisal third (InT) with 179.22 N. The conventional veneer with the finish line at the gingival level (CoV) displayed higher value with 404.07 N than any type of partial veneer. Tukey HSD post hoc analysis demonstrated statistically significant differences among all pairwise group comparisons (*p* < 0.05). Different superscript letters indicate statistically significant differences among groups.

**Figure 2:**
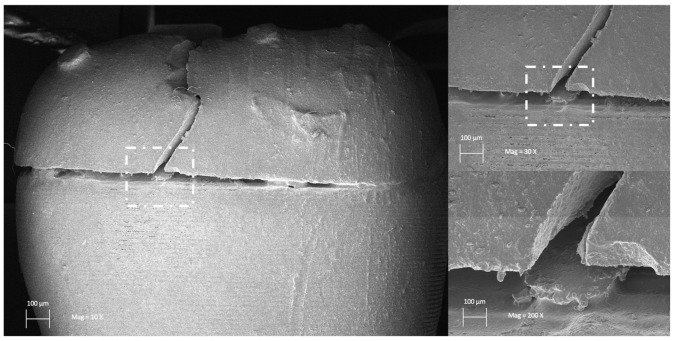
Representative scanning electron microscopy (SEM) image of a partial veneer with margin in the incisal third (InT) with 10×, 30× and 200× magnification.

**Figure 3:**
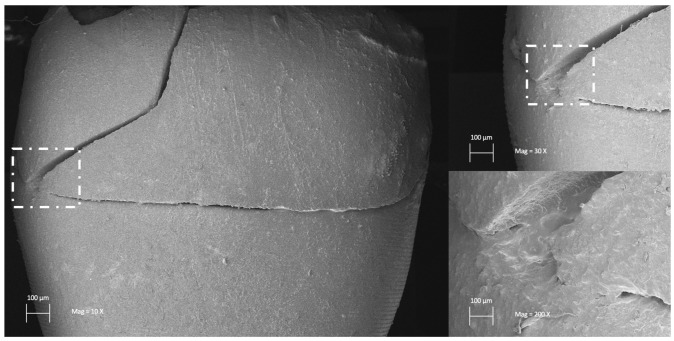
Representative scanning electron microscopy (SEM) image of a partial veneer with margin in the middle portion of the middle third (MmT) with 10×, 30× and 200× magnification.

**Figure 4:**
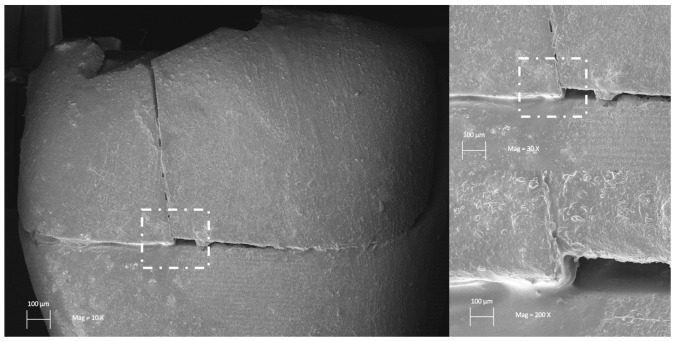
Representative scanning electron microscopy (SEM) image of a partial veneer with margin in the lower portion of the middle third (LmT) with 10×, 30× and 200× magnification.

**Figure 5:**
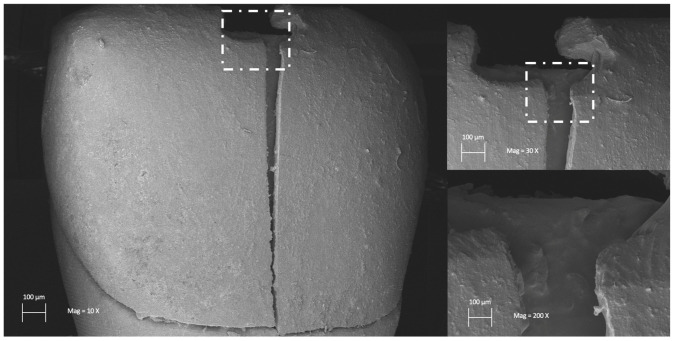
Representative scanning electron microscopy (SEM) image of a conventional veneer with margin at the gingival level (CoV) with 10×, 30× and 200× magnification.

Representative scanning electron microscope (SEM) images of fracture samples at 10×, 30× and 200× magnification are shown in Figure 2, Figure 3, Figure 4 and Figure 5. The fracture surfaces were analyzed from a fractographic perspective. Notably, InT (Figure 2) and MmT (Figure 3) displayed greater number of irregular cracks, while LmT (Figure 4) and CoV (Figure 5) displayed fewer and more uniform crack lines.

## 4. Discussion

This study compared the fracture resistance of artificially aged 3D-printed partial veneers with different preparation designs to that of conventional veneers. All specimens were fabricated from the same nanoceramic-filled resin, a material specifically developed for veneer restorations, using a completely digital workflow that involved computer-aided design and 3D printing. The statistical analysis led to rejection of the first null hypothesis, which proposed that no differences would be found in fracture resistance among the three partial veneer designs and the conventional veneer, since significant differences were detected among the groups (*p* < 0.001). The partial veneer groups, including the incisal third (InT), middle portion of the middle third (MmT), and lower portion of the middle third (LmT), demonstrated significantly lower fracture resistance values (InT: 179.22 N; MmT: 266.92 N; LmT: 279.86 N) compared with the conventional veneer group, in which the finish line was positioned at the gingival level (CoV: 404.07 N). The second null hypothesis, which stated that no differences would exist among the three 3D-printed partial veneer designs, was also rejected because significant differences were observed between the InT group and both the MmT and LmT groups.

The veneers were fabricated using a nanofilled ceramic resin material (Envision Rodin 3D Resin, Pac-Dent Inc., Brea, CA, USA) developed specifically for dental veneer applications. The manufacturer supplies this material as part of a complete system that includes the nanoceramic resin, adhesive, glaze, etching gel syringe, veneer resin cement, disposable applicators, mixing wells, and dispensing tips for the etching, bonding, and cementation procedures; the system has a reported shelf life of 24 months. The manufacturer recommends the use of its proprietary bonding and cementation components for veneer placement. Additionally, the Phrozen Sonic Mini 8K and Formlabs Form 4B have been validated by the manufacturer for producing restorations with this nanofilled ceramic resin. In the present study, the veneers were printed using one of these validated printers, the Phrozen Sonic Mini 8K, and were cemented following the manufacturer’s recommended protocol with the corresponding etching gel, adhesive, and resin cement to support optimal material performance.

Partial veneers have become a popular treatment option among clinicians, and recent data have demonstrated favorable outcomes. A recent multicenter retrospective study evaluated 79 partial laminate veneers placed on maxillary anterior teeth in 31 patients over an eight-year period. The restorations were assessed by calibrated clinicians using the United States Public Health Service criteria, and the cumulative survival rates were 100% after 1 year, 95.9% after 5 years, and 61.4% after 8 years. The authors concluded that partial laminate veneers demonstrated good long-term survival rates [27]. Furthermore, the literature includes case reports describing the successful use of partial ceramic veneers to mimic natural dentition with minimal tooth preparation while fulfilling patients’ esthetic and functional demands [28,29,30]. Given their popularity and favorable outcomes, partial veneer restorations with different designs were evaluated in the present study.

In the present study, the veneer restorations underwent artificial aging with thermal cycles. The use of thermal cycling has been validated in the literature, and 10,000 thermal cycles have been reported to simulate approximately 1 year of clinical service [31]. Several studies evaluating the fracture resistance of dental restorations have used a similar number of thermal cycles before fracture load testing [32,33,34]. Therefore, based on this well-established protocol, the restorations in the present study were subjected to 10,000 thermal cycles before being loaded to failure.

No studies are currently available evaluating the fracture resistance of 3D-printed partial labial veneers. However, the results of the present study are consistent with previous investigations evaluating ceramic veneers fabricated using other techniques, such as milling. A recent study compared the fracture resistance of conventional labial veneers with margins at the gingival level with three partial veneer designs: one with the margin in the incisal third and two with margins at different locations in the middle third. The study evaluated four brands of translucent zirconia and found that, regardless of the zirconia brand, conventional veneers with margins at the gingival level demonstrated higher fracture resistance than all partial veneer designs. The authors concluded that equigingival finish lines are recommended for restorations subjected to high mechanical demands [35]. Another study compared two partial veneer designs, with margins in the middle and incisal thirds, against conventional veneers with margins at the gingival level. The restorations were fabricated from fully crystallized CAD/CAM lithium disilicate, artificially aged with 10,000 thermal cycles, and loaded until fracture. The results showed that conventional veneers had higher fracture resistance than both partial veneer designs. The authors concluded that the fracture resistance of CAD/CAM lithium disilicate veneers is influenced by the extent of surface coverage and that conventional veneers demonstrate greater resistance to fracture [36]. Similarly, the present study compared conventional veneers with margins at the gingival level with different partial veneer designs fabricated from 3D-printed nanoceramic resin. The findings followed the same trend, with conventional veneers demonstrating higher fracture resistance than partial veneers.

The fractographic analysis displayed that the shorter the veneer, the higher the number of cracks and more irregular patterns. This trend suggests that the extent of restorative coverage may influence both fracture resistance and crack propagation patterns. Restorations with greater surface coverage may distribute loading forces more evenly across the bonded interface and supporting substrate, resulting in higher fracture resistance and more regular crack patterns. Conversely, smaller partial veneers may present reduced bonding surface area and greater stress concentration, which may explain their lower fracture resistance and the presence of more numerous and irregular cracks [37,38,39].

The present in vitro study has several limitations that should be acknowledged. First, printed resin dies were used instead of natural teeth. However, the resin used in the present study has a tensile strength of 61 MPa, which is within the range reported for natural dentin, 44 to 97.8 MPa [40,41]. Similar resins have also been used in previous studies evaluating the fracture resistance of dental ceramics [42,43]. Moreover, a recent study comparing natural teeth and resin die materials for fracture testing of zirconia crowns found no significant differences between resin dies and natural teeth, and the authors concluded that resin dies are a viable option for crown cementation and fracture load testing [44].

A limitation of the present study is that microstructural characterization, filler distribution analysis, and degree of conversion of this novel 3D-printed resin material were not evaluated. Future studies should assess these properties in greater depth, as these analyses would provide additional information on how changes in the material microstructure may influence its mechanical behavior, particularly its fracture resistance. Another limitation was the evaluation of only a butt-joint incisal preparation design. Future studies should assess other incisal edge designs, such as palatal overlap and feather-edge preparations. In addition, only one nanoceramic-filled resin material was evaluated; therefore, future studies should compare 3D-printed, milled, and conventionally fabricated resin veneer restorations. Future studies should also evaluate whether different finish margin designs, such as knife-edge or vertical margins, influence the fracture resistance of the restoration. Moreover, this study evaluated veneers fabricated only for maxillary central incisors, which may limit the generalizability of the findings to the broader anterior dentition. Because lateral incisors and canines differ in crown morphology, bonding surface area, and functional loading patterns, their fracture behavior may not fully correspond to that observed in central incisors. Future studies should therefore include other anterior teeth, particularly lateral incisors and canines, to provide a more comprehensive understanding of the clinical performance of these restorations. Lastly, future studies should include other artificial aging methods in addition to thermal cycling, such as artificial chewing, as this may provide a more clinically relevant simulation of the effects of masticatory forces before fracture testing of the restorations.

## 5. Conclusions

Based on the findings of this in vitro study, the following conclusions were drawn:

1. The fracture resistance of 3D-printed nanoceramic-filled resin veneers was influenced by the extent of surface coverage.
2. Partial-coverage veneers with margins located closer to the gingival level demonstrated higher fracture resistance than those with more incisal margins.
3. Conventional veneers with margins at the gingival level demonstrated higher fracture resistance than partial veneers.

## Figures and Tables

**Table 1:** Fracture resistance of 3D-printed partial and conventional veneers for maxillary central incisor.

Type of Restoration	Number of Samples	Load for Fracture (SD), Newtons
Partial veneer with margin in the incisal third (InT)	15	179.22 (±11.67) ^a^
Partial veneer with margin in the middle portion of the middle third (MmT)	15	266.92 (±13.32) ^b^
Partial veneer with margin in the lower portion of the middle third (LmT)	15	279.86 (±9.71) ^c^
Conventional veneer with margin at the gingival level (CoV)	15	404.07 (±11.53) ^d^

Note: Different superscript letters indicate significant difference (*p* < 0.05).

**Table 2:** Descriptive statistics for fracture resistance (N).

	Group 1 (InT)	Group 2 (MmT)	Group 3 (LmT)	Group 4 (CoV)
Mean	179.22	266.92	279.86	404.07
Standard error (SE)	3.01	3.44	2.50	2.97
Shapiro–Wilk (*p* value)	0.584	0.219	0.862	0.093
Range	161.13–201.21	247.92–288.41	261.65–294.48	388.04–420.89

Note: Shapiro–Wilk test indicated normal distribution in all groups (*p* > 0.05). Abbreviations: InT, Partial veneer with margin in the incisal third; MmT, Partial veneer with margin in the middle portion of the middle third; LmT, Partial veneer with margin in the lower portion of the middle third; CoV, Conventional veneer with margin at the gingival level.

## Data Availability

The original contributions presented in the study are included in the article, further inquiries can be directed to the corresponding author.
